# A multicenter randomized controlled trial evaluating the effect of small stitches on the incidence of incisional hernia in midline incisions

**DOI:** 10.1186/1471-2482-11-20

**Published:** 2011-08-26

**Authors:** Joris J Harlaar, Eva B Deerenberg, Gabrielle H van Ramshorst, Harold E Lont, Ed CMH van der Borst, Willem R Schouten, Joos Heisterkamp, Helena C van Doorn, Huib A Cense, Frits Berends, Hein BAC Stockmann, Wietske W Vrijland, Esther CJ Consten, Reyer T Ottow, Peter MNYH Go, John J Hermans, Ewout W Steyerberg, Johan F Lange

**Affiliations:** 1 Department of Surgery, Erasmus University Medical Center, Rotterdam, The Netherlands; 2 Department of Surgery, Vlietland Ziekenhuis, Schiedam, The Netherlands; 3 Department of Surgery, St. Elisabeth Ziekenhuis, Tilburg, The Netherlands; 4 Department of Gynaecology, Erasmus MC, Rotterdam, The Netherlands; 5 Department of Surgery, Rode Kruis Ziekenhuis, Beverwijk, The Netherlands; 6 Department of Surgery, Rijnstate Ziekenhuis, Arnhem, The Netherlands; 7 Department of Surgery, Kennemer Gasthuis, Haarlem, The Netherlands; 8 Department of Surgery, Sint Franciscus Gasthuis, Rotterdam, The Netherlands; 9 Department of Surgery, Meander MC, Amersfoort, The Netherlands; 10 Department of Surgery, Groene Hart Ziekenhuis Gouda, The Netherlands; 11 Department of Surgery, Antonius Ziekenhuis Nieuwegein, The Netherlands; 12 Department of Radiology, Erasmus MC, Rotterdam, The Netherlands; 13 Department of Public Health, Erasmus MC, Rotterdam, The Netherlands

## Abstract

**Background:**

The median laparotomy is frequently used by abdominal surgeons to gain rapid and wide access to the abdominal cavity with minimal damage to nerves, vascular structures and muscles of the abdominal wall. However, incisional hernia remains the most common complication after median laparotomy, with reported incidences varying between 2-20%. Recent clinical and experimental data showed a continuous suture technique with many small tissue bites in the aponeurosis only, is possibly more effective in the prevention of incisional hernia when compared to the common used large bite technique or mass closure.

**Methods/Design:**

The STITCH trial is a double-blinded multicenter randomized controlled trial designed to compare a standardized large bite technique with a standardized small bites technique. The main objective is to compare both suture techniques for incidence of incisional hernia after one year. Secondary outcomes will include postoperative complications, direct costs, indirect costs and quality of life.

A total of 576 patients will be randomized between a standardized small bites or large bites technique. At least 10 departments of general surgery and two departments of oncological gynaecology will participate in this trial. Both techniques have a standardized amount of stitches per cm wound length and suture length wound length ratio's are calculated in each patient. Follow up will be at 1 month for wound infection and 1 year for incisional hernia. Ultrasound examinations will be performed at both time points to measure the distance between the rectus muscles (at 3 points) and to objectify presence or absence of incisional hernia. Patients, investigators and radiologists will be blinded during follow up, although the surgeon can not be blinded during the surgical procedure.

**Conclusion:**

The STITCH trial will provide level 1b evidence to support the preference for either a continuous suture technique with many small tissue bites in the aponeurosis only or for the commonly used large bites technique.

**Trial registration:**

Clinicaltrials.gov NCT01132209

## Background

The median laparotomy is frequently used by abdominal surgeons to gain rapid and wide access to the abdominal cavity with minimal damage to nerves, vascular structures and muscles of the abdominal wall. However, incisional hernia remains the most common complication after median laparotomy, with reported incidences varying between 2-20%[1-5]. Even higher incidences up to 30-35% have been reported in obese and aortic aneurysm patients [6-10]. Incisional hernia can cause discomfort, impair quality of life or result in serious life-threatening conditions, such as incarceration or strangulation of the bowel [5]. Median laparotomies and incisional hernias have been subject of investigation for a long period of time already. Although a lot is known about patient related risk factors and suture materials, technical risk factors such as suture techniques have not been investigated thoroughly [5,11,12].

For prevention of incisional hernia, many clinical trials and meta-analyses have demonstrated that a mass closure technique with a simple running suture is the best option to close a midline incision. A mass closure technique with a running suture is also easier and quicker to perform than layered techniques with interrupted sutures [5,12-14]. Furthermore, the use of slowly resorbable suture material compared with non-resorbable suture material decreases the incidence of incisional hernia, and it also lowers the incidence and intensity of postoperative pain and wound infection [12,15,16].

### Suture length to wound length ratio and small bites

Several authors have stated that a suture length to wound length ratio (SL:WL) of four or more must be achieved, since a lower ratio is associated with an increased rate of incisional hernia [7,17-20]. It has often been recommended to place continuous stitches more than 10 mm from the wound edge in combination with a long stitch length [19,21-28]. A long stitch is the result of a large bite with the largest portion of fascia possible, aiming to increase tensile strength and to decrease the risk of fascial dehiscence. However, long stitches have been associated with high rates of both wound infection and incisional hernia [17,29,30]. A long stitch length may be associated with higher risks of wound infection due to an increase in the amount of necrotic tissue within the wound. In experimental studies, the long stitch length has been found to compress or cut through soft tissue included in the stitch [31,32]. The risk of incisional hernia may be higher because the stitch tends to slacken, which allows wound edges to separate.

Small stitches, placed 4-6 mm from the wound edge, only cut through the aponeurosis and not through the rectus abdominis muscle. Recent experimental data show that the small bites technique results in stronger wounds and faster healing than the routine large bite technique [33]. Our experiments in a porcine model showed a 47% increase in breaking strength when small bites were used compared to the routine technique [32]. A recent randomized of randomised clinical study by Millbourn et al. reported a decrease of incidence of incisional hernia of 70% 18% to 5.6%, p < 0.001) and a decrease of 50%, (10.2% to 5.2%, p = 0.020) of wound infection [34]. These results are very promising with regard to the prevention of incisional hernia and wound infection. The benefits of this technique need to be confirmed in a multicenter double-blinded randomized controlled trial.

In daily practice, most surgeons in the Netherlands use the large bite technique with large suture distances. With large bites, SL:WL ratio depends on the thickness of the abdominal wall including the muscles, the bite size, the number of stitches and the traction on the sutures during suturing. With large bites, an unanswered question remains with regard to how the SL:WL ratio of 4 should be reached. With a low traction force, fewer stitches are needed, but the slacking effect during the postoperative period may influence results.

With small stitches, SL:WL ratio is mostly dependent on the number of stitches. There is no sufficient evidence to prefer one suture closure technique over the other in order to prevent incisional hernia and fascia dehiscence.

### Objective

The objective of the STITCH trial (Suture Techniques to reduce the Incidence of The inCisional Hernia) is to compare the small bites technique decribed by Millbourn et al. with a standardized large bites technique.

The overall objective of the study is reduction of the incidence of the most frequent complication of abdominal surgery, i.e., incisional hernia. We hypothesize that the small bites technique will result in a significant reduction of the incidence of incisional hernia, which may lead to a reduced morbidity and a better quality of life for patients and a significant reduction of costs.

Primary endpoint will be incisional hernia occurrence within one year after surgery, either clinically and/or ultrasonographically detected. Secondary endpoints include postoperative complications, in particular surgical site infection, burst abdomen and wound pain in the first postoperative month.

## Methods/Design

### Trial Design

The STITCH trial has been designed as a prospective, multicenter, double-blind, randomized controlled trial, in which the large bites technique will be compared with the small bites technique.

### Participants

Patients scheduled for an elective abdominal operation through a midline incision will be asked for informed consent at the outpatient clinic or in hospital on the day preceding the day of surgery. Also, emergency laparotomies can be included in this trial if the patient is able to sign the informed consent. We intend to investigate the efficacy of the small bites technique in all risk groups. This also includes oncological gynaecological patients in centers with at least 50 median laparotomies a year.

#### Inclusion criteria

• Signed informed consent

• Laparotomy through a midline incision

• Age 18 years or older

#### Exclusion criteria

• Previous incisional hernia or fascial dehiscence with secondary healing after a midline incision

• Abdominal surgery through a midline incision within the last three months

• Pregnancy

Since the STITCH trial is an intervention study, it is not considered desirable to combine this trial with other intervention studies. In case of non-intervention (registration) studies, it will be judged on individual basis whether it is suitable and ethically correct to include a patient in both the STITCH trial and in another study. Patients will be included in the STITCH trial in combination with one other trial (registration trials only), provided that it is possible to organize the informed consent and the follow up in a proper way for the individual patient for both trials.

### Registration procedure

Included patient are registrated before surgery in an online data base (designed and managed by HOVON data center, Rotterdam, the Netherlands,) after signed informed consent via the Internet via TOP (Trial Online Process; see http://www.stitchtrial.nl). The patient namecode, date of birth, name of caller, name of responsible physician, sex and eligible criteria will be registered. Every participating institution has its own login code.

### Randomisation procedure

The randomization process is started only 15 minutes before closure to prevent consequences due to the trial during the operation with the online TOP randomisation.

Patients will be randomized between closure with the large tissue bites technique or with the small tissue bites technique. Randomisation is stratified by center, and between surgeon or resident with a minimization procedure, ensuring balance within each stratum and overall balance. The randomization result will be given immediately by TOP. A confirmation email without randomization result will be send to the investigator.

Patients will be kept unaware of the type of closure until the endpoint of the trial. Surgeons or residents blinded for the procedure will perform out patient clinic controls. Postoperative ultrasonography will be performed by radiologists blinded for type of closure. The randomisation procedure, blinding and objectification of incisional hernia by ultrasound will provide the best possible data to support preference for the large bites technique or the small bites technique over the other for closure of the abdominal wall.

### Interventions

In this trial the large bites technique will be compared with the small tissue bites technique as developed in Sundsvall Hospital, Sweden [18]. In the first group, the conventional large bites technique will be applied with bite widths of 1 cm and intersuture spacing of 1 cm with the use of one PDS plus II loop with a 48 mm needle. In the second group, the small bites technique will be applied with bite widths of 0,5 cm and intersuture spacing of 0,5 cm with the use of PDS plus II 2-0 with a 31 mm needle. In the small bites technique, twice as many stitches will be placed per sutured cm, with a smaller needle and thinner suture material. In the Swedish hospital where the small bites techniques has been in use for many years, this combination proved the easiest and safest method to perform the small bites technique [18,34].

In both groups wound length is measured before closing of the fascia. After measument of the woundlength, the number of stitches is calculated. In the large bites technique at least one suture per cm wound length must be placed. In the small bites technique at least two sutures per cm wound length must be placed. The number of stitches is counted by the assistant during closure.

In both arms, suturing is initiated at both ends of the incision towards the middle where an overlap will be created of at least 2 cm. The remaining sutures will be measured and the suture length used for closure of the fascia and the SL:WL ratio will be calculated by the scrub nurse. In both arms, suture length to wound length ratios (SL:WL) of 4:1 are aimed at.

### Implementation

In every hospital the OR nurses the surgeons or gynecologists and residents are instructed before the start of the trial in the individual institution during presentations and demonstration movies. During at least the first five inclusions the study coordinator will be present in the OR before randomization to assist randomization and control the correct applying of the standardized techniques. For every included patient a form with the detailed closing protocol is added to the clinical chart. Only when the surgeon is familiar with both the techniques, the nurses with the counting and measuring of the stitches and suture material and the study, centers are allowed to run the trial. Also, for every included patient a form with the detailed closing protocol is added to the clinical chart. During the study unplanned audits are performed to control quality.

#### Outcome parameters

##### Primary outcome

• Primary outcome will be incisional hernia occurrence within one year after surgery, either clinically and/or ultrasonographically detected.

##### Secondary outcome

• Postoperative complications

• Pain

• Quality of life

• Cost effectiveness

We use the definition of the incisional hernia by the European Hernia Society: 'any abdominal wall gap with or without bulge in the area of a postoperative scar perceptible or palpable by clinical examination or imaging'. The classification made by the European Hernia Society is used [35]. The classification of incisional hernias: Incisional hernias will be classified according to their localization, size, reducibility and symptoms.

Discharge dates and complications will be registered. Patients who fail to keep their annual clinic appointment will be given the option of a further appointment at a more suitable date or a visit to their home if they cannot make it to the outpatient clinic. The following data will be gathered at different points in time:

##### Preoperative data

• Date of birth

• Length and weight

• Current smoker (Yes or No)

• Medical history (including chronic obstructive pulmonary disease (COPD), diabetes mellitus, cardiac disease, prior laparotomies)

• Preoperative radiotherapy or chemotherapy

• Preoperative or perioperative corticosteroids

• Previous abdominal operations

• Other abdominal wall hernias

• American Society of Anaesthesiologists (ASA) classification

• Width of linea alba (if preoperative Computed Tomography Imaging is available)

### Operation data

• Type of operation

• Suture length: wound length ratio

• Number of stitches

• Length of incision

• Closure time

• Blood loss

• Operation time

• Antibiotic prophylaxis

• Drains and location

• Thrombosis prophylaxis

• Pain medication

• Peroperative complications (intestinal lesions, bleeding, other)

• Epidural catheter

#### Postoperative data

• Blood transfusion

• Postoperative ventilation and duration

• Postoperative corticosteroids

• Postoperative radiation therapy

• Postoperative pain medication

• Postoperative ileus and duration

• Postoperative complications:

○ Centers for Disease Control criteria for Surgical Site Infection, according to the guidelines proposed by Mangram in 1999 [36] Appendix 1.

○ Wound haematoma: accumulation of blood in the wound area, which warrants surgical exploration and intervention.

○ Pulmonary infections

○ Ventilation problems

○ Re-admission and indication

○ VAS pain score until day 6 post operative

At 1 and 12 months, ultrasound imaging will be performed to examine the midline for any asymptomatic clinically not detectable incisional hernias. Size and location of any incisional hernias will be registered.

### Outpatient clinic follow up

• Outpatient clinic visit at 1 and 12 months

○ Incisional hernia

○ Wound infection

○ Seroma formation

○ Other wound problems

○ Other abdominal wall hernia

• Ultrasound at 1 and 12 months

• VAS pain scores and Quality of Life forms preoperatively (day of operation or the day before) and at 1,3, 6 and 12 months

#### Ultrasound examinations

During the 1 month and 1 year follow up an ultra sound examination will be performed to measure the distance between the rectus muscles at 3 point in the incision and check for incisional hernia. A specific score is used for the ultrasound examination. At ten points, which include 4 measurements of the distance between the rectus muscle, the quality of the scar in the abdominal wall is objectified. With this method the conclusion if there is an incisional hernia can also be made on the score list. In this list is controlled for:

An intact linea alba?

Bulging without Valsalva manouvre?

Bulging with Valsalva manouvre?

Distance between rectus muscles in scar on 1/3 cranial part in cm?

Distance between rectus muscles in scar on 1/3 caudal part in cm?

Maximum distance between rectus muscles in scar in cm?

Maximum distance between rectus muscles at place of bulging or defect in cm?

Is there a defect? If yes, the size of the defect and location

Is there fatty tissue in the defect?

Is there a bowel loop in the defect?

The radiologist is asked to make prints of every measurement and finding.

Quality of life will be assessed based on standardized Quality of Life forms including the EuroQol-5D and Short Form-36 before and at 1 month, 3 months, 6 months, and 12 months after surgery.

#### Economic evaluation

We will perform an ex-post economic evaluation in which a new suture technique using small bites is compared with the traditionally applied large bites technique, from a societal perspective. The economic evaluation will be performed in accordance with Dutch guidelines (Oostenbrink, 2004).

To measure the economic impact of the new suture technique using small bites the cost-effectiveness will be assessed by calculating the incremental cost-effectiveness ratio, defined here as the difference in average costs between both suture techniques divided by the difference in average effects. The primary outcome measure will be the costs per reduced incisional hernia within 1 year. Secondary, a cost-utility analysis will be performed using costs per quality adjusted life year (QALY) as outcome measure, using the EQ-5D.

Costs for all separate actions and time used by all individual health care professionals, and all other materials will be measured from a societal perspective for both bites techniques, which means that both direct medical costs (e.g. intervention costs, intramural and extramural medical costs) and indirect costs (absence from work, patient costs) will be included in the analysis.

For the most important cost items, unit prices will be determined by following the micro-costing method (Gold et al, 1996), which is based on a detailed inventory and measurement of all resources used. Resource costs arise within the hospital and consist of outpatient visits, inpatient days, use of the operation room, radiology examinations, blood tests, etc. Real medical costs will be calculated by multiplying the volumes of health care use with the corresponding unit prices. For instance, the calculation of the costs of both suture techniques will consist of detailed measurement of investments in manpower, equipment, materials, housing and overhead. The salary schemes of hospitals and other health care suppliers will be used to estimate costs per hour for each health care professional. Taxes, social securities and vacations will be included.

Data on effects (reduction of incisional hernia), costs (time costs of new suture technique and material and development costs) and savings (reduced health care use of patients without incisional hernia) will all be collected in this study. Data on treatment (hospitalisation) and follow-up consultations will be collected retrospectively from (electronic) patient charts and hospital administration. This data will be collected by health care professionals using a data-collection form. Information will collected on:

- length of hospital stay

- length of stay in ICU

- reinterventions

Data on extramural care, work absence and other patient costs will be gathered via questionnaires at each follow-up (1 and 12 months).

For a description of the calculation of the effect measures see paragraph 'outcome parameters'.

Discounting of future costs and effects is not relevant because of the limited time horizon of 1 year. When costs of a treatment are similar across subgroups, the absolute benefit determines the cost-effectiveness of a treatment for a specific subgroup.

Randomized controlled trials are designed to evaluate the effects of treatment at the group level, and cost-effectiveness is usually calculated for this group as a whole. There could however be substantial and relevant between subgroup variability. It is therefore common to consider subgroup specific effects of interventions. The subgroup specific cost-effectiveness will be estimated by first deriving a prognostic index, based on the predefined predictors of incisional hernia: abdominal aneurysm aorta (AAA), obesity, diabetes, COPD, corticosteroid usage, radiotherapy, cardiovascular disease, smoking, age, cancer, other abdominal wall hernias and collagen disorders.

### Sample size calculation

Millbourn et al. found a decrease in the incidence of incisional hernia from 18% to 5,6% in a randomized controlled trial [34]. In this trial, follow-up consisted of clinical instead of radiological examination for incisional hernia occurrence. In this trial, ultrasound examination will be used in order to be able to diagnose incisional hernia with higher sensitivity. It is expected that a relative decrease of the incidence incisional hernia after one year of 50% is reasonable. The mean reported one year incidence of incisional hernia in literature is 15%[1-5]. In order to reduce the mean incidence of incisional hernia from 15 to 7.5%, power calculations showed that two groups of 259 evaluable patients each are needed (power = 0.80, alfa = 0.05). Loss to follow-up is estimated at 10% of included patients. A total of 576 patients (2 × 288) will be included in the study to correct for loss to follow-up. Overall effects will be calculated adjusted for predictive baseline characteristics, which will lead to a higher statistical power.

### Statistical analysis

Descriptive statistics will include median and interquartile range for continuous variables, and absolute numbers (with %) for categorical variables. Randomized groups will be compared for imbalance without formal statistical testing. Analysis will be by intention-to-treat. Differences between randomized groups will be tested with appropriate statistical methods, including t-tests or Mann-Whitney tests for continuous variables (considering whether the normality assumption is rejected by the Kolmogorov-Smirnov test with Lilliefors correction test), and chi-square tests for categorical variables. The primary outcome (incisional hernia) will be analyzed with Kaplan-Meier analysis and a Cox regression analysis, to adjust for any loss to follow up between 30 days and 1 year after surgery. The primary analysis is a covariate adjusted Cox model, which includes the following predefined, well-establihed predictors of incisional hernia: abdominal aneurysm aorta (AAA), obesity, diabetes, corticosteroid usage, radiotherapy, COPD, smoking, age, cancer, inguinal hernia, cardiovascular disease and collagen disorders.

Subgroup effects will be assessed by tests of interaction to prevent overinterpretation of apparent differences in effectiveness. Quality of life data will be analyzed by paired T-tests, comparing baseline with follow-up measurements, and repeated measures analysis. A two-sided p < 0.05 will be taken to indicate statistical significance.

#### Monitoring

The Erasmus University Medical center is the sponsor of this trial. Adverse events are defined as any undesirable experience occurring to a subject during a clinical trial, whether or not considered related to the investigational intervention. All adverse events reported spontaneously by the subject or observed by the investigator or his staff will be recorded. A serious adverse event (SAE) is any untoward medical occurrence or effect that at any dose results in death; is life threatening (at the time of the event); requires hospitalization or prolongation of existing inpatients' hospitalization; results in persistent or significant disability or incapacity; is a new event of the trial likely to affect the safety of the subjects, such as an unexpected outcome of an adverse reaction, major safety finding from a newly completed animal study, etc. All SAEs will be reported to the accredited Medical Ethical Committee (MEC) that approved the protocol, according to the requirements of that MEC. Serious Adverse events are death and burst abdomen. Adverse Events are readmission and reoperations.

An independent data and safety monitoring committee will evaluate the progress of the trial and will examine safety parameters every 3 months. The committee can unblind the data whenever deemed necessary based on reported adverse events. All involved physicians will repetitively be asked to report any potential adverse events caused by the study protocol. These adverse events will be listed and discussed with the monitoring committee. The monitoring committee can ask for a full report in order to discuss a specific adverse event. A copy of this report will be sent to the central ethics board and to the involved physicians. All deceased patients will be evaluated by the safety committee for cause of death and possible trial related serious adverse effects. Every death will be reported to the central ethics board and the local ethics board. The Data Safety Monitoring Board will consist of an epidemiologist/statistician and two independent surgeons.

#### Ethics

This study will be conducted in accordance with the principles of the Declaration of Helsinki and 'good clinical practice' guidelines. The Medical Ethical Committee of the Erasmus University Medical Center Rotterdam has approved the protocol. The Ethical Committees of the participating centers are applied for local feasibility. Prior to randomization, written informed consent will be obtained from all patients.

## Discussion

A major issue in all suture studies is standardisation of technique. In a multicenter trial it is difficult to achieve standardisation because many surgeons and residents will contribute in this trial. The benefit of a large group of participants is that the results will be representable for daily practice.

In this trial two major parameters have been standardized: the difference between small and large bites and the amount of stitches per running cm of wound resulting in an appropriate SL:WL ratio.

In daily practice, most surgeons use the large bite technique with large suture distances. With large bites, SL:WL ratio depends on the thickness of the abdominal wall including the muscles, the bite size, the number of stitches and the traction on the sutures during suturing. With large bites there is an unanswered question under which conditions an optimal SL:WL ratio of 4 should be reachable. With low traction on the suture fewer stitches are needed, but the slacking effect during the postoperative period will influence the results. For this reason in a RCT on suture techniques it is necessary to standardize the amount of stitches per centimetre of wound length.

## Conclusion

The STITCH trial is a multicenter randomized trial (trialregister: http://clinicaltrials.gov/ct2/show/NCT01132209) comparing the costs and effectiveness of a standardized small tissue bites suture technique with a standardized large tissue bites technique in midline incisions. This trial will provide the surgical society the evidence needed to optimize a surgical technique used to prevent common surgical complications.

## Competing interests

The Erasmus MC "Doelmatigheids Onderzoek grant 2008" and Johnson and Johnson Medical BV, the Netherlands, Investigator Initiated Clinical Research Funding Grant (09-107) have financially supported this trial.

## Authors' contributions

JJH drafted the manuscript. EBD, GHR, EWS and JFL co-authored the writing of the manuscript. All other authors participated in the design of the study during several meetings and are local investigators at the participating centres. All authors edited the manuscript and read and approved the final manuscript.

## Appendix 1

### Criteria for defining a Surgical Site Infection (SSI)

#### Superficial Incisional SSI

Infection occurs within 30 days after the operation and infection involves only skin or subcutaneous tissue of the incision and at least one of the following:

1. Purulent drainage, with or without laboratory confirmation, from the superficial incision.

2. Organisms isolated from an aseptically obtained culture of fluid or tissue from the superficial incision.

3. At least one of the following signs or symptoms of infection: pain or tenderness, localized swelling, redness or heat and superficial incision is deliberately opened by surgeon, unless incision is culture-negative.

4. Diagnosis of superficial incisional SSI by the surgeon or attending physician.

Do not report the following conditions as SSI:

1. Stitch abscess (minimal inflammation and discharge confined to the points of suture penetration).

2. Incisional SSI that extends into the fascial and muscle layers (see deep incisional SSI).

### Deep Incisional SSI

Infection occurs within 30 days after the operation if no implant is left in place or within 1 year if implant is in place and the infection appears to be related to the operation and

infection involves deep soft tissue (e.g., fascial and muscle tissue) of the incision and at least one of the following:

1. Purulent drainage from the deep incision but not from the organ/space component of the surgical site.

2. A deep incision spontaneously dehisces or is deliberately opened by a surgeon when the patient has at least one of the following signs or symptoms: fever (> 38°C), localized pain, or tenderness, unless site is culture negative.

3. An abscess or other evidence of infection involving the deep incision is found on direct examination, during re-operation, or by histopathological or radiological examination.

4. Diagnosis of a deep incisional SSI by a surgeon or attending physician.

#### Notes

1. Report infection that involves both superficial and deep incision sites as deep incisional SSI.

2. Report an organ/space SSI that drains through the incision as a deep incisional SSI.

### Organ/Space SSI

Infection occurs within 30 days after the operation if no implant is left in place or within 1 year if implant is in place and the infection appears to be related to the operation and infection involves any part of the anatomy (e.g., organs or spaces), other than the incision, which was opened or manipulated during an operation and at least one of the following:

1. Purulent drainage from drain that is placed through a stab wound into the organ/space.

2. Organisms isolated from an aseptically obtained culture of fluid or tissue in the organ space.

3. An abscess or other evidence of infection involving the organ/space that is found on direct examination, during reoperation, or by histopathologic or radiologic examination.

4. Diagnosis of a deep organ/space SSI by a surgeon or attending physician.

## Pre-publication history

The pre-publication history for this paper can be accessed here:

http://www.biomedcentral.com/1471-2482/11/20/prepub
